# Effective combination of lymphatico‐venous anastomosis and negative pressure wound therapy for lymphocyst: A Case Study

**DOI:** 10.1111/jog.14300

**Published:** 2020-05-28

**Authors:** Ayano Shimono, Hisashi Sakuma, Shiho Watanabe, Hikaru Kono

**Affiliations:** ^1^ Department of Plastic and Reconstructive Surgery Yokohama Municipal Citizen's Hospital Yokohama Japan; ^2^ Department of Plastic and Reconstructive Surgery Tokyo Saiseikai Central Hospital Tokyo Japan; ^3^ Department of Plastic and Reconstructive Surgery National Center for Child Health and Development Tokyo Japan

**Keywords:** lymph node excision, lymphatic cyst, negative pressure wound therapy, surgical anastomosis, vaginal cancer

## Abstract

Lymphorrhea and lymphocysts are complications that occur after lymph node dissection or biopsy and are difficult to treat. Conventional treatments for lymphocysts are not always effective. For instance, lymphatico‐venous anastomosis has a limited treatment efficacy when the cyst wall is thickened, and negative pressure wound therapy is limited by the installation site and longer treatment times. To overcome these individual shortcomings, we aimed to assess whether a combination of both interventions would be effective. In this study, we report the application of a lymphatico‐venous anastomosis combined with negative pressure wound therapy for treating bilateral inguinal lymph nodes and pelvic lymph node dissection following treatment of vaginal cancer. Short‐term improvements were observed with no recurrence of lymphocysts at 1‐year follow‐up.

## Introduction

Among the complications that can occur after lymph node dissection or biopsy, lymphorrhea and lymphocysts routinely develop but are often difficult to treat.^1^ The reported frequency of lymphorrhea and lymphocysts during abdominal and pelvic lymph node dissection is 30–70%^2^ and 1–49%, respectively.^3^ To prevent lymphorrhea during lymph node dissection, it is useful to cauterize lymphatic vessels under direct view. If lymphorrhea continues, hypoalbuminemia and electrolyte abnormalities will occur.^4^, ^5^

We present a case wherein a combination of lympho‐venous anastomosis (LVA) and negative pressure wound therapy (NPWT) was used to treat bilateral inguinal lymphocysts after pelvic lymphadenectomy for vaginal cancer. Short term improvements were observed with no signs of recurrence 1 year later.

### Case report

A 70‐year‐old woman was diagnosed with stage I vaginal cancer (pT1N0M0) in July 2018 after complaining of irregular bleeding, which began in February 2018. She was treated with laparoscopic radical hysterectomy, bilateral oophorectomy, pelvic lymphadenectomy, as well as shallow and deep inguinal lymphadenectomy in July 2018. Postoperative diagnosis was stage I vaginal cancer (squamous cell carcinoma, keratinizing type, pT1N0M0). Postoperatively, lymphocysts formed bilaterally in the inguinal region, and the patient was referred to us 3 weeks after surgery.

**Figure 1:**
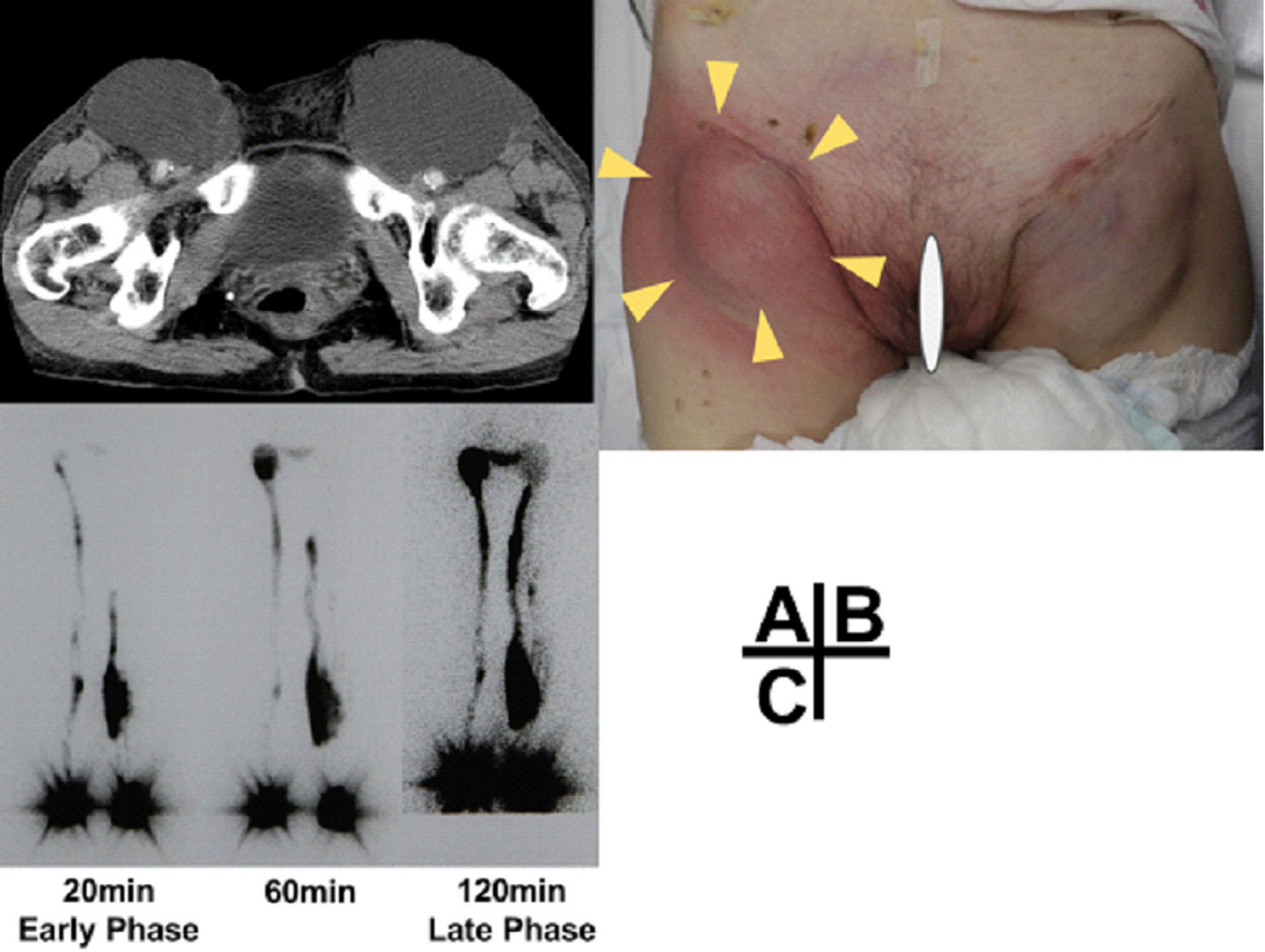
(a) Lymphocysts formed in the bilateral inguinal region found on computed tomography. (b) External appearance of lymphocysts formed in the bilateral inguinal region with the right lymphocyst showing signs of infection (pointers). (c) Lymphoscintigraphy results: Dermal backflow can be observed in the left lower leg during the early phase, while Radio Isotope leakage to bilateral inguinal lymphocysts and stasis in the collecting lymphatic vessel can be seen in the images taken during the late phase.

At the first visit, preoperative computed tomography revealed lymphocysts measuring 7‐cm bilaterally on the groin, with no internal mass (Fig. 1a). Approximately 2 weeks after the initial visit, signs of infection appeared around the right lymphocyst (Fig. 1b). The lymphocyst showed elevated leukocytes (9810/μL) and c‐reactive protein (5.3 mg/L). A transparent, yellow liquid was drained after puncturing the infected lymphocyst. Bacterial culture of the lymphocyst puncture showed negative results. The infection persisted, which necessitated hospitalization of the patient for 2 days following the culture collection. An intravenous antibiotic (1‐g Cefazolin/8‐h) was administered. Lymphoscintigraphy showed dermal backflow in the left lower leg with bilateral inguinal lymphocysts during the early phase, and stasis in the collecting lymphatic vessels during the late phase (Fig. 1c).

**Figure 2:**
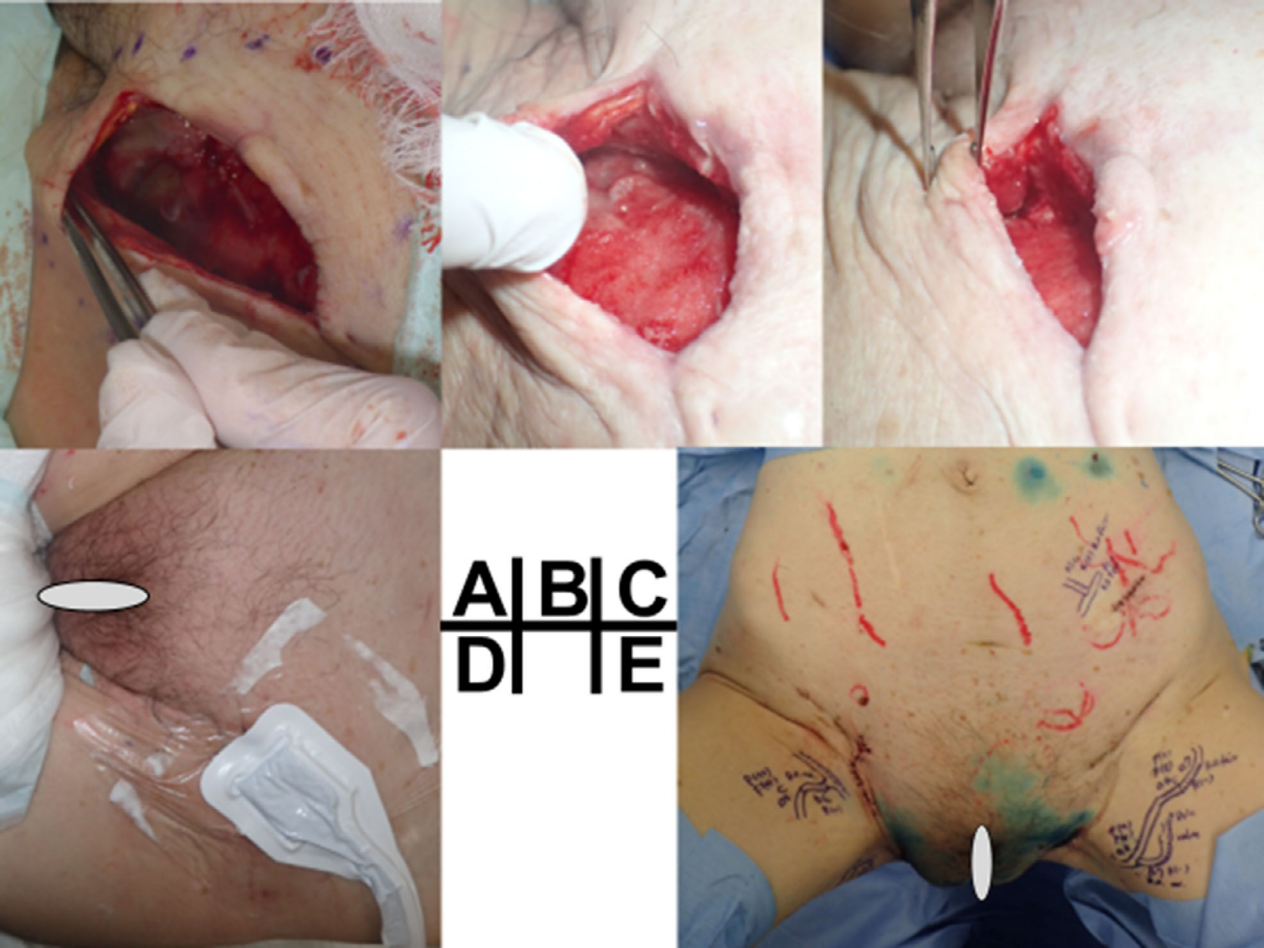
(a) Negative Pressure Wound Therapy (NPWT) Day 1. The thickened septum inside the left groin lymph cyst is incised inferiorly. (b) NPWT Day 8. Developing granulation tissue can be seen while the cyst wall remains.(c) NPWT Day 12. The granulation has developed further, and the wound is observed to have shrunk in size. The inner cyst wall has been removed. (d) External appearance of a negative pressure wound therapy device (RENASYS) is attached. The sponge is about half the size of the cyst. (e) We performed bilateral lymphocyst puncture and five lymphovenous anastomoses (three left femoral end‐to‐end, one left lower leg end‐to‐side, and 1 right femoral end‐to‐end) under general anesthesia.

**Figure 3:**
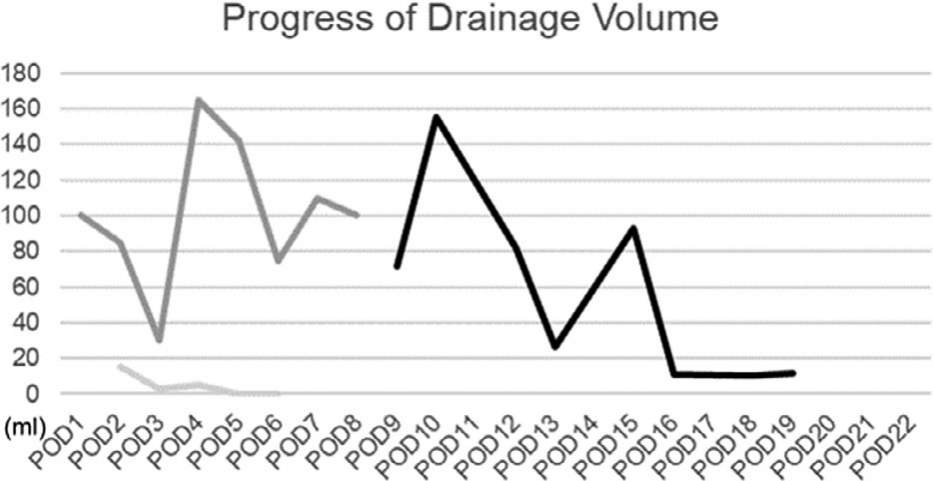
Drainage has steadily decreased since Negative Pressure Wound Therapy (NPWT) was started. NPWT was terminated 13 days after commencement. (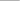), left, (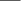), RENASYS and (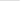), right

After the infection subsided, we performed bilateral lymphocyst puncture and five lymphovenous anastomoses (three left femoral end‐to‐end, one left lower leg end‐to‐side, and one right femoral end‐to‐end) under general anesthesia, on the 7th day of hospitalization (Fig. 2e). A 10‐Fr JVAC drain (ETHICON) was placed in each of the left and right lymphocysts, prior to closure. Drainage decreased steadily, and the drain placed in the right lymphocyst was removed after 6 days. The amount of fluid drained from the left side also decreased. However, subcutaneous lymph retention was observed despite the maintenance of negative pressure drainage. Manual drainage was performed to drain approximately 100 cc/day, and the drain was removed after 7 days. On the 10th postoperative day, we opened the left inguinal lymphocyst under local anesthesia and applied NPWT at −80 mmHg (RENACYS, Smith & Nephew). Before applying the negative pressure dressing, we opened the previous surgical wound and removed the internal cyst wall with a scalpel. Calcium alginate dressing (Sorbsan, ALCARE) was applied to cover the exposed vaginal ligament and the femoral vein. A sponge shaped to about half the size of the cyst was inserted into the wound before performing NPWT. The septum inside the cyst was observed to gradually separate and was extracted. At the same time, granulation formed within the wound and wound contraction were observed (Fig. 2a–d). NPWT was terminated 13 days after installation. Drainage had steadily decreased since the beginning of NPWT (Fig. 3). A subcutaneous drain was inserted and removed 3 days after wound closure, and suture was removed 12 days after NPWT termination.

At 1‐year follow‐up, despite mild edema in the lower abdomen and vulva, the lower limb edema showed significant improvement and lymphocysts had not recurred.

This research was deemed ethical by our institution's IRB review policy. Informed consent was obtained from the study participant, including consent to participate and to publish the findings.

## Discussion

Lymphocysts are treated by either of the two ways: (i) controlling the amount of lymphatic fluid leakage, and (ii) reducing the empty space created by lymph node dissection. Control of lymphorrhea is non‐surgically achieved through restricting fat intake and subcutaneous administration of a somatostatin analog. Surgically, lymphorrhea is controlled by ligation of the involved lymphatic vessels using fibrin glue to coat tissues suspected of lymphorrhea, and an LVA was performed. 1, 3, 4, 6 To reduce the empty space non‐surgically, sclerotherapy (isodine, minomycin, ethanol, or OK‐432 solutions) is used. Surgically, the empty space can be covered using a myocutaneous flap with the anterior sacral muscle or the sternocleidomastoid muscle.^4^

We performed LVA to drainage the lymphocyst and reduce pressure in the lymphatic vessels. Iwamoto *et al*. reported the first case of LVA for treating postoperative pelvic lymphocysts.^6^ They reported that LVA reduces the cysts by indirectly reducing the incoming lymph flow. However, Todokoro *et al*. reported that only 6 of 11 lymphocysts were improved using an LVA alone.^3^ These studies suggest that an LVA is effective only when an anastomotic lymphatic vessel is the dominant lymphatic channel. In addition, we ascertained that the effect of LVA alone is limited in cases of thickened non‐collapsible cysts. In our patient, the capsule of the lymphocyst was fibrotic and thickened due to infection, and the cyst wall could not be removed only by LVA. Although LVA could reduce the lymphorrhea, the lymphocyst was not treated adequately. Moreover, debriding the cyst wall mixed with normal tissue in a three‐dimensional mosaic necessitates the simultaneous drilling of the unaffected tissues. In our case, the femoral arterial vein was near the lymphocyst, which posed a challenge for debridement. Therefore, we decided to use NPWT. When there is a wound with space, we put sponge inside the space and cover the wound with a film. Cut the film partially directly above the sponge, then attach a suction tube on the film defect. Inside the wound will be negative pressure, which is reported to promote wound healing.^2^, ^4^, ^5^, ^7^, ^8^, ^9^ Usually, we change sponge and disinfect the wound twice a week. We found that NPWT can safely remove the cyst wall. When the sponge is removed, the tissue around the sponge sticks on the sponge, and is removed together.

When Fleischmann *et al*. reported on NPWT as a treatment for lymphorrhea for the first time in 1993, a negative pressure of −200 mmHg was recommended for the collapse of capillary lymphatic vessels.^10^ More recently, other groups reported the use of negative pressure ranging from −75 to −125 mmHg.^2^, ^5^, ^7^, ^8^ In these cases, NPWT was terminated after 35–80 days.

In 2018, Yuan *et al*. reported on the world's first successful rabbit model for the treatment of lymphorrhea.^9^ A lymphorrhea model involving bilateral inguinal regions of both male and female rabbits was treated with either NPWT or gauze dressing. All rabbits treated using NPWT were cured within 11 days, whereas none of those treated with gauze dressing were cured in that period. The authors suggest that NPWT can successfully treat lymphorrhea by exerting pressure on the enlarged lymphatic vessels and promoting lymphorrhea to decrease lymph flow stagnation and thus, improve leg edema.

We found that another possible mechanism of action of NPWT is the ability to slowly and mechanically debride the cyst wall and promote suction of the lymphatic fluid by refreshing the wound. This is believed to reinvigorate the wound healing mechanism and promote wound contraction. Although there are many reports of NPWT being used for treating lymphorrhea, there are only a few reports on the application of NPWT for treating lymphocysts for longtreatment periods.^2^

LVA and NPWT have advantages and disadvantages in the treatment of lymphatic cysts. LVA reduces lymphatic flow into the cysts but shows poor therapeutic effect when the cyst wall is thickened, as the cyst wall disturbs wound adaptation. NPWT removes lymphatic cysts and promotes wound healing, but it is insufficient to reduce lymphatic flow into the cysts. However, it is limited to local intervention with a prolonged treatment duration. Considering these factors, we chose a combination of both treatments and observed a synergistic therapeutic effect. This method is effective when LVA alone does not stop lymphorrea and lymph cysts form a thick septum, which require a slow debridement, but the wound is located nearby a risky structure which make us hesitate to debridement. We suggest to perform LVA first to reduce lymphatic flow into the cysts. Post‐operative day 1, confirm the bleeding from the wound is stopped, and attach NPWT.

We reported a case in which lymphocysts improved in a short period of time with the combination of LVA and NPWT without any postoperative recurrence at the 1‐year follow‐up. In cases where the cyst wall is thickened due to secondary infection, it may not be possible to treat the cyst with LVA alone, and it is important to reactivate the wound‐healing mechanisms by applying NPWT in tandem.

## Disclosure

None declared.
